# Parallel processing in the honeybee olfactory pathway: structure, function, and evolution

**DOI:** 10.1007/s00359-013-0821-y

**Published:** 2013-04-23

**Authors:** Wolfgang Rössler, Martin F. Brill

**Affiliations:** Behavioral Physiology and Sociobiology (Zoology II), Biozentrum, University of Würzburg, Am Hubland, 97074 Würzburg, Germany

**Keywords:** Antennal lobe, Glomeruli, Projection neurons, Mushroom bodies, Multi-unit recording

## Abstract

Animals face highly complex and dynamic olfactory stimuli in their natural environments, which require fast and reliable olfactory processing. Parallel processing is a common principle of sensory systems supporting this task, for example in visual and auditory systems, but its role in olfaction remained unclear. Studies in the honeybee focused on a dual olfactory pathway. Two sets of projection neurons connect glomeruli in two antennal-lobe hemilobes via lateral and medial tracts in opposite sequence with the mushroom bodies and lateral horn. Comparative studies suggest that this dual-tract circuit represents a unique adaptation in Hymenoptera. Imaging studies indicate that glomeruli in both hemilobes receive redundant sensory input. Recent simultaneous multi-unit recordings from projection neurons of both tracts revealed widely overlapping response profiles strongly indicating parallel olfactory processing. Whereas lateral-tract neurons respond fast with broad (generalistic) profiles, medial-tract neurons are odorant specific and respond slower. In analogy to “what-” and “where” subsystems in visual pathways, this suggests two parallel olfactory subsystems providing “what-” (quality) and “when” (temporal) information. Temporal response properties may support across-tract coincidence coding in higher centers. Parallel olfactory processing likely enhances perception of complex odorant mixtures to decode the diverse and dynamic olfactory world of a social insect.

**Electronic supplementary material:**

The online version of this article (doi:10.1007/s00359-013-0821-y) contains supplementary material, which is available to authorized users.

## Introduction

Olfaction is of paramount importance for the survival of most animal species. The question of how the highly complex molecular space within the olfactory world is encoded into neuronal activity and processed to finally result in adaptive behavioral responses has attracted many studies over many years. Social insects have to deal with a particularly complex odor world in the context of food search, general orientation in the environment, communication by pheromones, and the detection of species-specific recognition cues like chemical profiles on the insect cuticle (Hölldobler and Wilson 1990; Hölldobler 1999; Sandoz et al. 2007; Smith et al. 2009; Wyatt 2010; Hansson and Stensmyr 2011; Martin et al. 2011).

Processing of information along parallel pathways (within and across sensory modalities) represents an important feature in most sensory systems (Young 1998; Rauschecker and Scott 2009). The most prominent examples for parallel processing within a sensory modality come from research in the vertebrate visual system. Magno‐ (M) and parvocellular (P) pathways of the lateral geniculate nucleus provide input to segregated layers of the primary visual cortex (Callaway 2005; Lennie and Movshon 2005). Within these pathways, visual information is subdivided into color (M) and spatio‐temporal (P) information and was shown to be important for visual perception (Livingstone and Hubel 1988; Merigan and Maunsell 1993). Parallel pathways within the visual system were also found in insects, for example within the optic ganglia, the medulla, and segregated pathways to higher-order centers in the mushroom bodies (MBs) (Ribi and Scheel 1981; Fischbach and Dittrich 1989; Strausfeld et al. 2006; Paulk et al. 2008, 2009). In vertebrate auditory systems, parallel pathways may code different parameters underlying different tasks, and this is expected to support the speed and accuracy of sensory information processing (Knudsen et al. 1987; Nassi and Callaway 2009). In the insect auditory system, information is relayed from auditory receptor neurons to interneurons that transfer information via separate streams either preferentially mediating sound recognition or the detection of directional information (e.g., Helversen and Helversen 1995). Compared to visual, auditory, or somatosensory systems, parallel processing in the olfactory system—for example the input–output relationships within chemotopic maps of olfactory glomeruli—is much less understood (Galizia and Rössler 2010; Sandoz 2011; Brill et al. 2013). Analysis of the neuroanatomical characteristics, physiological role, and behavioral relevance of parallel sensory information streams within the olfactory system is crucial for understanding olfactory coding and perception in general.

Recent reports in vertebrates indicate that the olfactory bulb output via mitral/tufted cells can be divided into distinct channels of parallel olfactory information (Fukunaga et al. 2012; Igarashi et al. 2012; Payton et al. 2012). Anatomically, in insects multiple parallel antennal-lobe (AL) output tracts have been identified including a dual olfactory pathway in Hymenoptera comprising two distinct AL-output pathways to higher-order olfactory centers in the mushroom bodies (MBs) and lateral horn (LH) (Abel et al. 2001; Kirschner et al. 2006; Zube et al. 2008; Galizia and Rössler 2010). Social behavior in honeybees heavily relies on olfactory recognition and communication (e.g., Winston 1987; Seeley 1995; Slessor et al. 2005). The honeybee, therefore, has become a key model system for the study of olfactory processing, perception, and learning for many years (e.g., Menzel and Giurfa 2001; Galizia and Rössler 2010; Sandoz 2012; Menzel 2012).

A recent review by Galizia and Rössler (2010) integrated comparative aspects of anatomically *parallel olfactory pathways* across insects and suggested hypotheses for potential modes of segregated and/or parallel processing along these pathways. The present review focuses on *parallel olfactory processing* with special emphasis on the dual olfactory pathway in the honeybee (*Apis mellifera*). Physiological data from very recent studies in the honeybee strongly support parallel processing in this system and, in addition, have triggered new hypotheses on the potential role of temporal coding within and across olfactory tracts. We used the large body of data available for the specialized system in the honeybee to provide a focused review on this species. This is also aimed to stimulate future approaches on parallel processing in this and other olfactory systems. We further integrated a discussion of recent work on the evolutionary origin of a dual olfactory tract within the Hymenoptera.

For a more general review on basic anatomical and physiological features of the peripheral and central levels of the insect olfactory system (including the honeybee olfactory system), we like to refer to comparative reviews within the past 3 years on insect olfaction (e.g., Galizia and Rössler 2010; Hansson and Stensmyr 2011; Sandoz 2011; Martin et al. 2011; Nawrot 2012).

## Anatomical features of a dual olfactory pathway

**Fig. 1:**
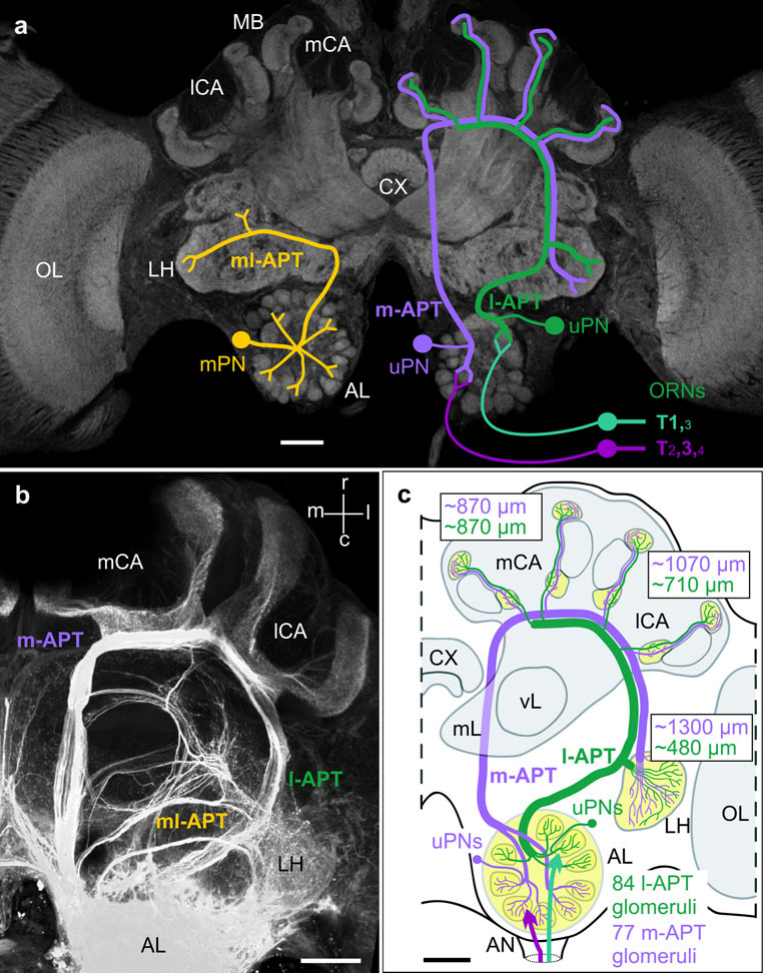
Anatomical features of parallel olfactory systems in the honeybee brain with special emphasis on a dual olfactory pathway from the antennal lobe (*AL*) to higher-order centers in the mushroom bodies (*MB*) and lateral horn (*LH*). **a** Schematic drawings of individual projection neurons (*PN*) from different antennal-lobe protocerebral tracts (*APT*) superimposed on a confocal image of the honeybee brain. *Right brain half* schematic drawings of a medial-tract uniglomerular PN (m-APT, uPN) and a lateral-tract PN (l-APT, uPN). *Left brain half* schematic drawing of a multiglomerular PN (mPN) that projects to the lateral horn (LH) only via the medio-lateral tract (ml-APT). The sensory input from olfactory receptor neurons (ORNs) to l- and m-APT associated glomeruli via four sensory-input tracts (T1-4) is schematically indicated on the right side. The size of the tract numbers depicts the dominance of different tracts in the two AL hemilobes. Adapted and modified with permission from Rössler and Zube (2011). **b** Projection view of an anterograde mass-fill of all APTs. Projection of the two major m- and l-APT from the AL to the medial and lateral MB calyces (mCA, lCA) of the MB and the LH. Three m- and l-APT (1–3) branch off the m-APT and innervate the lateral protocerebrum. Adapted and modified with permission from Kirschner et al. (2006). **c** Schematic overview of the dual olfactory pathway in the honeybee. ~84 glomeruli in the upper half of the AL are innervated by l-APT PNs that target the LH first and then the lCA and mCA. The m-APT originates from ~77 glomeruli in the lower half of the AL and projects to the mCA and lCA first before it targets the LH. The approximate distances of axonal trajectories via the m- and l-APT pathway to the three targets are indicated in *green* (l-APT) and *magenta* (m-APT). Adapted and modified with permission from Kirschner et al. (2006). *AN* antennal nerve, *CX* central complex, *OL* optic lobes, *mL* and *vL* medial and vertical lobes of the MB. *Scale bars* in **a**–**c** 100 μm

In the honeybee AL, ~160 olfactory glomeruli function as primary processing units for incoming olfactory information from olfactory receptor neurons (ORNs) housed in olfactory sensilla on the antenna (Galizia et al. 1999; Kirschner et al. 2006). After preprocessing in local AL circuits via local interneurons, the olfactory information is relayed from individual glomeruli via two separate uniglomerular projection-neuron (PN) output pathways to the MB calyx and the LH (Mobbs 1982; Abel et al. 2001; Kirschner et al. 2006; Galizia and Rössler 2010; Rössler and Zube 2011) (Fig. 1). In addition, at least three tracts formed by axons of multiglomerular PNs (PNs that innervate many glomeruli) project to the lateral protocerebrum, in particular the LH. The distinct neuroanatomical characteristics of a dual uniglomerular PN pathway targeting the two MB calyces and LH in each brain hemisphere in opposite sequence represent a most striking feature in the olfactory pathway of Hymenoptera (Kirschner et al. 2006; Galizia and Rössler 2010; Rössler and Zube 2011; Nishikawa et al. 2012) (Fig. 1). Regarding the innervation of glomeruli by PNs of both output pathways in the honeybee, the AL is divided into two about equally sized hemilobes containing glomeruli innervated by PNs with axons projecting either via the medial or the lateral antennal-lobe protocerebral tract (m- and l-APT). The m-APT comprises axons from ~410 PNs innervating ~77 glomeruli in the lower AL hemilobe, whereas the l-APT contains axons from ~510 PNs innervating ~84 glomeruli in the upper AL hemilobe (Kirschner et al. 2006; Rybak 2012).

Glomeruli in the honeybee AL are associated with four different olfactory receptor neuron (ORN) sensory-input tracts (T1–T4) and can be grouped into four distinct input-tract-specific glomeruli clusters (Abel et al. 2001; Kirschner et al. 2006). Whereas the l-APT is mainly supplied by PNs receiving input from T1 glomeruli (and to a small extent by T3 glomeruli), the m-APT is mainly supplied by T3 glomeruli (and to a small extent by T2 and T4 glomeruli) (indicated in Figs. 1, 3). Almost all AL glomeruli receive sensory input from the main type of olfactory sensilla on the antenna—the sensilla placodea (Kelber et al. 2006; Nishino et al. 2009). Thus, olfactory input received by ORNs in sensilla placodea is fed into both the m- and l-APT hemilobes. The projections of ORNs axons from other types of olfactory sensilla, in particular sensilla basiconica and sensilla trichoidea, are currently investigated by selective tracing studies (Kropf et al. 2012).

Axons of m-APT PNs target the medial and lateral MB calyces first and then the LH, whereas axons from l-APT PNs target the LH first, then the lateral and medial MB calyces forming a system reminiscent of two opposing delay-line-like neuronal circuits in each brain hemisphere (Galizia and Rössler 2010; Brill et al. 2013) (Fig. 1a, c). The terminal projections of PNs from both tracts within the MB-calyx lip remain largely segregated in concentric layers forming an outer layer of exclusively m-APT PN projections, an inner core with mainly l-APT PN projections, and an intermediate zone with largely overlapping input between both (Kirschner et al. 2006; Zube et al. 2008; Nishikawa et al. 2012). Similarly, within the multimodal (visual, olfactory) basal ring of the MB calyx, olfactory input from both tracts is organized into two distinct concentric layers. Within the LH, the target areas of l- and m-APT PNs are largely segregated with a region of overlap in the central part of the LH (Kirschner et al. 2006; Zube et al. 2008; Nishikawa et al. 2012).

## Past and present hypotheses for odorant processing along a dual olfactory pathway

The honeybee dual olfactory pathway has been subject to various studies asking the question whether the two projection neuron pathways represent two segregated information streams or serve parallel processing (Galizia and Rössler 2010; Sandoz 2011; Nawrot 2012).

The special anatomical characteristics of two very distinct AL-output tracts in the honeybee provide a unique opportunity for combined neuroanatomical, neurophysiological, and behavioral approaches to investigate parallel olfactory processing. To prove the biological significance for parallel olfactory processing along a dual olfactory pathway, it is crucial to know whether the two PN tracts in the honeybee code odorants in a “dual segregated” fashion (different odorants in different tracts) like in pheromonal and general-odorant subsystems in moths (Martin et al. 2011) or in flies (Schlief and Wilson 2007), or in a “dual parallel” fashion (similar input, differential feature extraction) as outlined by Galizia and Rössler (2010). The most important next step, therefore, was to establish simultaneous recordings from PNs of both tracts in same individuals to ask whether the two PN tracts process different or similar odorants (Brill et al. 2013). In the latter case, it is important to test whether different parameters are extracted from same odorants in a sense that different information is extracted in parallel from multiple sensory maps (Young 1998). The different neurophysiological approaches that were used to test this hypothesis will be the topic of the next section.

In addition to the hypothesis on parallel/segregated processing outlined above, we want to put forward a new hypothesis for temporal odorant coding along the dual olfactory pathway, which is derived from the specific anatomical characteristics of the dual olfactory pathway in the honeybee (Kirschner et al. 2006). The two PN pathways can be viewed as a delay-line-like system with opposing polarity and convergent output via PN axonal collaterals at three distinct target points—the medial and lateral MB calyces, and the LH (Fig. 1c). If we consider the differences in the distances of m- and l-APT PN axonal trajectories, we can expect differences in the spike-arrival times along m- and l-APT PNs at the three target points (Kirschner et al. 2006) (see distances indicated in Fig. 1c). The distances along the m- and l-APT to the medial MB calyx are similar, whereas the distances to the lateral MB calyx and LH are different between the two tracts. If we assume typical conduction velocities of 20–25 cm/s as found in the honeybee brain (Oleskevich et al. 1997), simultaneously evoked action potentials along both pathways should arrive about synchronously at the medial MB calyx, but with a delay of ~2 ms in the lateral MB calyx and a delay of ~4 ms in the LH (Fig. 1c and model in Fig. 3). A model of two opposing delay lines appears attractive as various studies of temporal coding in insect olfactory systems have demonstrated synchronization in PN activities (Laurent et al. 1996; Wehr and Laurent 1996; Stopfer et al. 1997, 2003; Lei et al. 2002; Perez-Orive et al. 2002, 2004; Ito et al. 2009; Riffell et al. 2009a, b, 2013; Gupta and Stopfer 2012). Furthermore, the unique electrical properties of MB target neurons (Kenyon cells, KCs) as shown in the locust and cockroach suggest that synaptic input to KCs provides an ideal substrate for coincidence detection by KCs from synchronized input of PNs (Laurent 2002; Perez-Orive et al. 2002, 2004; Cassenaer and Laurent 2007; Demmer and Kloppenburg 2009; Tabuchi et al. 2012). Whether the two opposing delay-line-like PN circuits in the dual olfactory pathway of the honeybee employ a temporal code that may serve coincidence detection by KCs critically depends on temporal coding aspects of m- and l-APT PNs and their convergence on KCs.

## Parallel processing via a dual olfactory pathway: neurophysiological evidences

### Intracellular recordings of individual projection neurons

The dual olfactory pathway in the honeybee brain with its obvious segregation into two AL-output pathways to higher-order centers in the MBs and LH represents one of the best known examples of parallel olfactory pathways within the main olfactory system (Galizia and Rössler 2010). Various neurophysiological approaches have been used to investigate processing of olfactory information within the two pathways. The first systematic studies on olfactory processing by PNs belonging to the medial and lateral pathways were done by independent, sequential intracellular electrophysiological recordings from individual PNs recorded in the AL of different individuals followed by tracer injections to identify the tract-specific PN morphologies (Sun et al. 1993; Abel et al. 2001; Müller et al. 2002; Krofczik et al. 2008). Müller et al. (2002) found that l-APT PNs code odorants by spike-rates with broader odorant-tuning profiles compared to m-APT PNs. In addition, the authors found evidence that odorant information was on average conveyed faster by l-APT PNs compared to m-APT PNs. From these analyses of odorant-tuning properties and response latencies of individual PNs at relatively high odorant concentrations Müller et al. (2002), it can be concluded that the two populations of PNs may code similar odorants using neuronal strategies for processing different properties of the same stimulus. This study was followed by another study using pooled data from sequential intracellular recordings from l‐ and m‐APT PNs in different bees (Krofczik et al. 2008). The main result of this study was a difference in the two PN populations regarding mixture-coding properties. Mixture responses in m-APT PNs were dominated by the most effective compound (elemental representation), whereas l-APT PNs exhibited suppressed responses to mixtures, but not to single compounds (synthetic representation).

### Calcium imaging of glomerular activation

The fact that individual PNs from both tracts responded to similar odorants raised the question whether glomeruli in the two AL hemilobes receive largely similar (or redundant) olfactory input. This was recently addressed by two calcium-imaging studies of glomerular activation in the AL (Carcaud et al. 2012; Galizia et al. 2012). The two groups used two different preparation techniques for sequential recording of odorant-evoked activation of glomeruli in the l- and m-APT hemilobes of the AL. Both studies used bath application and bulk loading with calcium-sensitive dyes. This technique is believed to preferentially monitor calcium fluctuations caused by ORN activity in AL glomeruli. Using stimulation with a selected panel of odorants, the results of both studies led to a similar conclusion: sensory input to l- and m-APT glomeruli within both AL hemilobes appears remarkably redundant. The study by Carcaud et al. (2012) revealed slight coding preferences for chain length and functional group of the odorant stimulus between m- and l-APT associated glomeruli, whereas the study by Galizia et al. (2012) found slight differences in the response strengths of calcium activations between the two subsystems. Another calcium-imaging study in the ant *Camponotus floridanus* monitored glomerular activation of projection neurons in response to stimulation with colony odors by selectively loading PNs with calcium indicator (Brandstaetter and Kleineidam 2011). The authors concluded that the two m- and l-APT associated AL subsystems known from anatomical studies in this ant (Zube et al. 2008) either receive similar sensory input or sensory input is locally distributed across both AL hemilobes. In the honeybee, analyses of the l- and m‐APT PN pathways by calcium-imaging techniques were extended to the level of PN-output synapses by selective loading of PNs with calcium indicators and sequential imaging of calcium activation in PN synaptic boutons in the MB calyx of different individuals (Yamagata et al. 2009). Odorant-evoked calcium activation in m-APT PN boutons indicated more broadly tuned and less concentration-dependent response properties, whereas in l-APT PNs responses were more narrowly tuned and more or less concentration invariant for the panel of odorants used in this study. Similar as in the study by Krofczik et al. (2008) this study revealed higher levels of mixture suppression in l-APT PNs compared to m-APT PNs. The partly contradicting results regarding odorant processing features at high odorant concentrations in the study by Yamagata et al. (2009) compared to results from intracellular PN recordings by Müller et al. (2002) may be caused by differences in the recording position (PN axon at the exit of the AL versus terminal boutons in the MB calyx) and the different activity measures that were used (sodium driven action potentials in axons versus presynaptic calcium activation in synaptic boutons). Furthermore, calcium activation in PN boutons in the MB calyx very likely is influenced by local MB circuits such as GABAergic feedback networks (Grünewald 1999; Perez-Orive et al. 2002; Gupta and Stopfer 2012) and/or neuromodulatory influences (Galizia and Kreissl 2012; Grünewald 2012; Himmelreich and Grünewald 2012).

### Simultaneous multi-unit recordings from multiple projection neurons of both olfactory tracts

To conclude, all above-mentioned electrophysiological and imaging studies give support to the hypothesis that the honeybee dual PN pathway serves parallel olfactory processing of similar odorants. However, the studies partly suffer from low sample rates of recorded PNs or animals, relatively low numbers of different odorant stimuli used, as well as limited numbers of stimulus repetitions due to very restricted time windows for intracellular recordings and in calcium-imaging experiments. In addition, poor temporal resolution of in situ fluorimetric calcium measurements did not allow detection of potentially relevant differences in temporal response properties between PNs of both tracts. Most importantly, however, none of these studies recorded PN activity from both olfactory information streams simultaneously from PNs of both tracts in individual bees under exactly the same stimulus conditions.

**Fig. 2:**
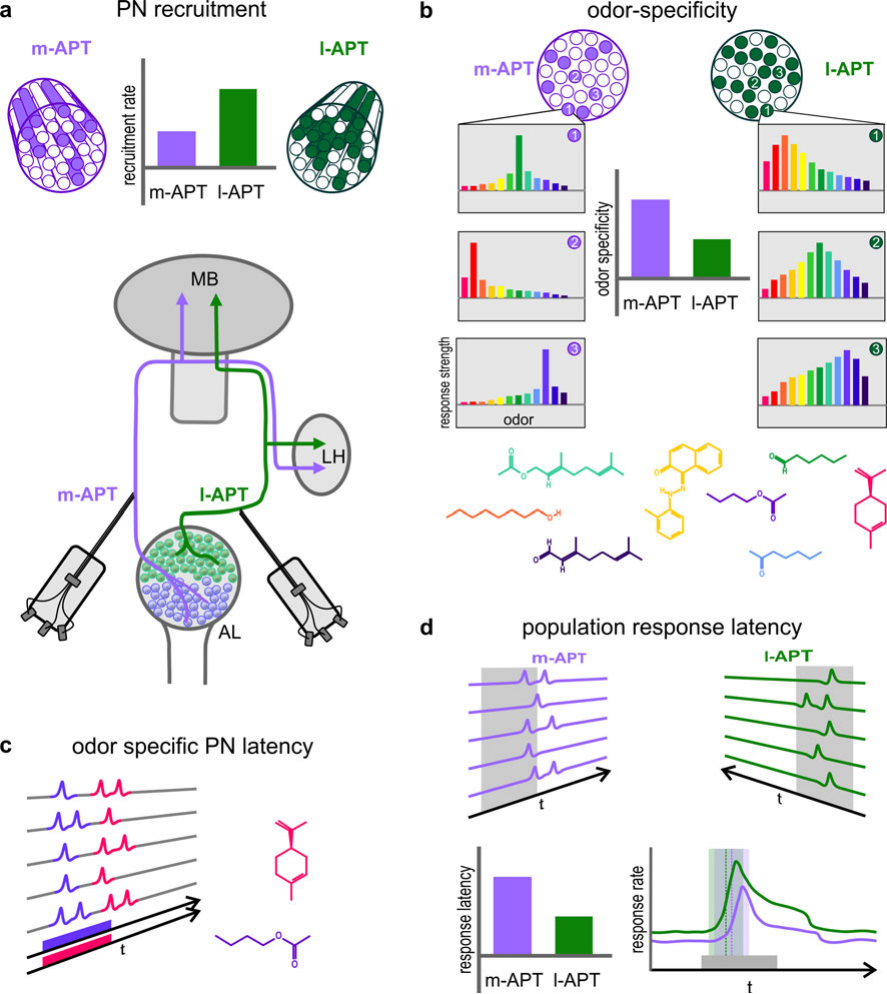
Parallel odorant processing in the honeybee dual olfactory pathway. Summary of main results based on multi-unit electrophysiological recordings by Brill et al. (2013) comprising projection neuron (*PN*) responses to 17 different floral, pheromonal, combined floral–pheromonal as well as biologically relevant odorants (see text for details). PNs were recorded simultaneously from the l- and m-APT using multi-unit recordings with thin-wire electrodes. **a** Differences in PN recruitment: odorant stimulations elicited activity in ~50 % of all l-APT PNs compared to ~30 % in m-APT PNs. The recording position of multi electrodes is depicted in the scheme below. **b** Differences in odorant specificity: individual m-APT PNs respond with high odorant specificity, whereas l-APT PNs show a broader (generalistic) odorant tuning. The numbers depict individual PNs. Different odorants are indicated by *different colors*. **c** Differences in response latency: different odorants elicited different response latencies in ~30 % of individual PNs suggesting latency coding of odorant identity. Two different odorants and the PN responses to them are *color coded*. **d** The average response latencies of l- and m-APT PNs differ with the l-APT responding significantly faster (lower graph on the left hand side). Despite this difference, the PN population responses of m- and l-APT PNs are largely overlapping (*lower graph on the right-hand side*)

To overcome these limitations, simultaneous recordings from large numbers of PNs of both tracts with high temporal precision were needed to further investigate parallel olfactory processing in this model system. This technical challenge was recently solved in a study by Brill et al. (2013) by establishing a novel technique for simultaneous multi-unit electrophysiological recordings from PNs of both tracts using customized thin-wire electrodes (modified after Mizunami et al. 1998; Strube-Bloss et al. 2011, 2012) and appropriate (template-matched) spike-sorting and analysis tools (Nawrot et al. 2003; Meier et al. 2008) (Fig. 2). The spike activity was recorded directly and simultaneously from multiple PNs (up to five simultaneously recorded PNs on each side) in the l- and m-APT. To make sure that the activity originated from these two populations of PNs, the recording position of the multi electrodes in the output tracts above the AL was verified by double labeling of the electrode positions as well as post-recording staining and 3D reconstruction of the output tracts after successful dual-tract recordings (Brill et al. 2013). A relatively large panel of 17 different odorants was used in this study including floral, pheromonal, and combined floral/pheromonal odorants as well as complex natural mixtures such as beeswax, dead bees, honey, and brood comb at the appropriate hive temperature (~34 °C). Using these techniques, simultaneous dual-tract recordings were achieved that lasted over several hours allowing many odorant presentations with a high number of stimulus repetitions. A comparison of the odorant stimuli used by Brill et al. (2013) with those used by other investigators is provided by Table 1 in supplementary materials.

The most important finding of the study by Brill et al. (2013) is that simultaneously recorded PNs of both tracts had widely overlapping response profiles in response to all odorants tested. This is in accordance with the results of calcium imaging of the olfactory input (Carcaud et al. 2012; Galizia et al. 2012) and fulfills a central requirement for a role of the two PN populations in parallel olfactory processing. Whereas l-APT PNs responded with ~14 ms shorter latencies (on average 170 ms after stimulus onset at odorant concentrations of 1:100) and broad odorant–response profiles (on average ~50 % recruitment rates) indicating generalized odorant coding properties, m-APT PNs responded with significantly longer latencies (on average ~184 ms) and had significantly higher odorant specificity (on average ~30 % recruitment rates) compared to l-APT PNs (Fig. 2). This was verified both at the level of simultaneously recorded PNs in individual bees as well as at the PN population level. The authors concluded that broadly tuned l-APT PNs deliver fast and more global information about the timing or temporal structure of an odorant stimulus, whereas m-APT PNs provide more specific information about odor identity. In analogy to the “what-” and “where-” subsystems in the vertebrate visual pathway (Mishkin et al. 1983; Merigan and Maunsell 1993; Milner and Goodale 2008), the two parallel subsystems in the honeybee olfactory pathway may provide “what-” (quality) and “when” (temporal) olfactory information (Fig. 2). The results of the multi-unit electrophysiology study by Brill et al. (2013) are highly suggestive that PNs of both APTs receive largely similar input within the panel of odorants used for stimulation. However, even though some of the odorants used were complex mixtures from the natural environment (hive odors, social odors, and floral odors), this set of odorants still has to be considered as rather limited compared to the complexity of the natural odor world. Therefore, it cannot be excluded that certain odorants may still be transferred via PNs of one APT only, especially considering that the large odor space bees are confronted with (Guerrieri et al. 2005; Schmuker and Schneider 2007; Haddad et al. 2008, 2010; Chen et al. 2011). Future experiments, therefore, will have to expand the neurophysiological analyses within the behaviorally relevant odor space to confirm whether the dual pathway operates as a parallel-processing system across all biologically relevant odors.

**Fig. 3:**
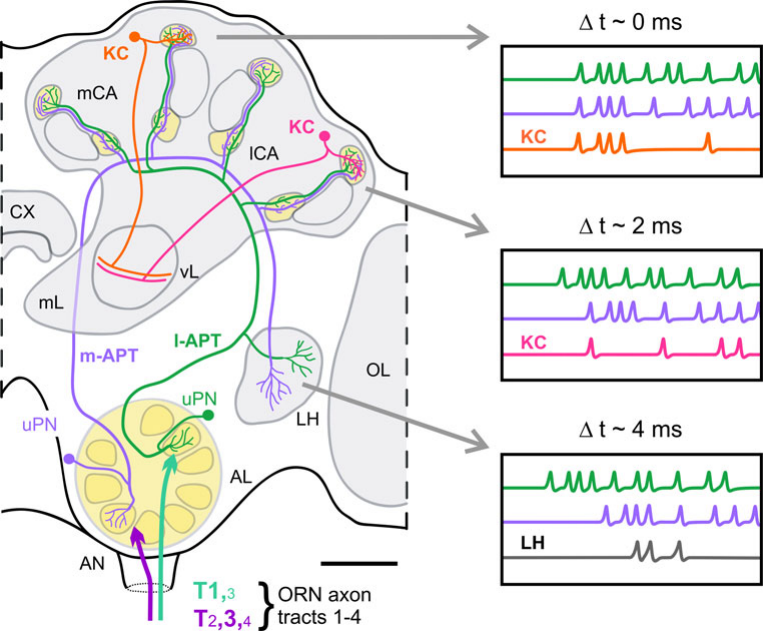
Model on a delay-line-like organization in the honeybee dual olfactory pathway based on anatomical and physiological findings (see text for details). Schematic drawings show individual medial and lateral antennal lobe protocerebral tract (*m-*, *l-APT*) uniglomerular projection neurons (*uPNs*). Schematic drawings of two individual Kenyon cells (*KC*) are included in the medial and lateral mushroom-body calyx (*mCA, lCA*) that receives convergent input from the two uPNs. The estimated differences in the delay of action potentials from m- and l-APT PNs at the mCA, lCA and lateral horn (*LH*) based on typical conduction velocities in the honeybee and differences in anatomical distances are indicated on *top of the boxes on the right-hand side* (see text for details). According to this model, differences in the delay of PN responses (Δ*t*) at the mCA and lCA lead to hypothetical differences in coincident activation of the KC in the mCA and lCA, as well as in a hypothetical postsynaptic LH neuron that receives convergent input from PNs of both tracts. *AN* antennal nerve, *AL* antennal lobe, *CX* central complex, *mL* medial lobe of the MB, *ORN* olfactory receptor neuron, *T1–4* sensory-input tracts 1–4, *vL* vertical lobe of the MB. *Scale bar* 100 μm

In ~30 % of the recorded PNs of both tracts, response latencies of individual m- and l-APT PNs were odorant dependent. PNs of the two pathways, on average, showed an overall difference in response latency with the l-APT PNs being faster than the m-APT PNs (Fig. 2c, d). Despite these differences in the responses of individual PNs, analyses at the population level found that PNs from both tracts show a substantial overlap in their temporal response profiles (Fig. 2d). This brings up the question whether temporal response profiles are relevant for temporal coding and potential across-tract coincidence detection by KCs in the medial and lateral MB calyces and/or postsynaptic neurons in the LH (Figs. 1, 3). The degree of temporal overlap of PN spike sequences within and across tracts (Fig. 2d) suggests that the dual olfactory pathway has the potential to promote across-tract coincidence and/or sparse coding at the level of KCs. Careful temporal correlation analyses of PNs recorded in same individuals are necessary to extract precise information about stimulus-dependent spike coincidences in PNs within and across tracts (Brill et al. 2013) (for further considerations, see “Conclusions and outlook” section and model in Fig. 3).

## Evolution of a dual olfactory pathway

The division of the AL-output tracts in a medial and lateral APT of uniglomerular PNs targeting the medial and lateral MB calyces and the LH in reverse order is not unique to the honeybee and was shown to be a common feature in Hymenoptera (Zube et al. 2008; Galizia and Rössler 2010; Rössler and Zube 2011; Nishikawa et al. 2012). In the ant *C. floridanus*, despite a much higher total number of olfactory glomeruli in the AL (~430 in the ant compared to ~160 in the honeybee) and a higher number of ORN sensory-input tracts (7 in the ant versus 4 in the honeybee), a very similar division of AL in two hemilobes with two about equally large populations of glomeruli associated with the l- and m-APT was found (Zube et al. 2008; Zube and Rössler 2008). Although the division in two uniglomerular PN populations is very obvious in bees and ants, this does not exclude that other insects that have only one tract of uniglomerular PNs to the MBs do not possess distinct subpopulations of PNs that may serve parallel olfactory processing within the same tract. Recently, in the cockroach two distinct subpopulations of PNs within the m-APT were shown to innervate two domains of glomeruli within the AL (Nishino et al. 2010, 2012). Simultaneous recordings from these two PN populations, however, are still lacking. In contrast to a single PN tract to the MBs with subpopulations of PNs, the unique anatomical features of a dual PN pathway in the honeybee and other Hymenoptera (Rössler and Zube 2011) may promote enhanced parallel-processing capabilities via delay-line-like circuits formed by the two opposing output tracts (Figs. 1, 3; and see “Conclusions and outlook” section).

Did a dual l- and m-APT PN pathway evolve within the Hymenoptera? Comparison across insect orders revealed that a dual uniglomerular PN pathway to the MBs and LH appears to be unique to Hymenoptera (Galizia and Rössler 2010). Results from a comparative neuroanatomical tracing study by Rössler and Zube (2011) show that a dual pathway from the AL to the MBs is present in social bees, basal and advanced ants, solitary wasps, and in one of two investigated species of plant-feeding sawflies (Symphyta), a basal group of plant-feeding Hymenoptera. A comparative study on two species of sawflies (*Neodiprion ventralis* and *N. autumnalis*; Diprionidae) (Dacks and Nighorn 2011) revealed “moth-like” characters within the AL and in output tracts with only a small l-APT to the LH and MB. The study by Rössler and Zube (2011) on two other species belonging to the Symphyta revealed that a prominent l-APT was present in *Diprion pini* (Diprionidae), but absent in *Athalia rosae* (Tenthredinidae). Further preliminary tracing studies in our laboratory indicate that a prominent dual pathway appears to be present in *Megalodontes* sp. (Megalodontesidae) and *Tenthredo cf. oliviaca* (Tenthredinidae) (W. Rössler, personal communication). This suggests that a dual olfactory pathway may have emerged within the group of basal, plant-feeding Hymenoptera (Dacks and Nighorn 2011; Rössler and Zube 2011). The evolutionary origin of a higher complexity in AL-output tracts in certain species within the Symphyta certainly needs further comparative investigations, in particular the question whether this may have occurred under specific ecological circumstances. In the same line, detailed behavior studies are needed to narrow down potential selective pressures that may have led to the evolution of multiple parallel olfactory pathways to the MBs and LH within the group of plant-feeding sawflies. One possibility may be the level of complexity in olfactory perception and communication in these species.

Does the presence of a dual olfactory pathway promote a social life style? We hypothesize that potential advances in olfactory processing via a dual olfactory pathway may represent a more general pre-adaptation for life styles with high demands on olfactory discrimination like advanced food searching or egg laying strategies (central place foraging, repetitive visits of feeding or egg laying sites, quality of the substrate for egg deposition, etc.), parasitoidism, as well as social communication and organization (Dacks et al. 2006, 2010; Dacks and Nighorn 2011; Rössler and Zube 2011). Comparative immunohistochemical studies by Dacks et al. (2006, 2010) indicate that the level of complexity in histaminergic local neurons and serotonergic systems in the AL showed a substantial degree of morphological modification within the Hymenoptera. To further test how changes in the olfactory system may have contributed to evolutionary transitions in life styles within the Hymenoptera, we certainly need more comparative neuroanatomical investigations correlated with life-history, ecological, and behavior data. To understand the role of parallel olfactory pathways, we need behavioral and physiological studies in the future to analyze olfactory capabilities in closely related species with and without a dual olfactory pathway to the MBs and LH.

The studies by Dacks and Nighorn (2011) and Rössler and Zube (2011) further suggest that the occurrence of a dual output pathway to the MBs appears to be independent from the presence of a high number of olfactory glomeruli in the AL and duplicated MB calyces. This was supported by the fact that a dual pathway was present in a species of sawflies with a rather small number of AL glomeruli (~30–40) and duplicated (although small) MB calyces, whereas it was absent or weekly expressed in another species of sawflies with a similarly small number of AL glomeruli and duplicated MB calyces (Dacks and Nighorn 2011; Rössler and Zube 2011). This led to the hypothesis that the origin of a dual PN pathway may be related to changes in the number and/or type of PN populations within the AL. Whether this is actually the case needs to be shown in comparative analyses of PN numbers and types in closely related species with and without a dual tract to the MBs. A developmental study in the honeybee (Groh and Rössler 2008) found that the m-APT synaptic target regions in the MB calyx develop slightly earlier compared to l-APT regions. This may reflect a difference in the developmental origin of the two PN populations, which is also supported by the fact that m- and l-APT PNs differ regarding the location of their soma clusters on the surface of the AL as shown for both the honeybee and the ant *C. floridanus* (Kirschner et al. 2006; Zube et al. 2008).

Did the evolution of a dual olfactory pathway promote the emergence of social life styles within the Hymenoptera? A comparative study by Farris and Schulmeister (2011) reported that the presence of large elaborate MB calyces appears, for the first time, in pre-social parasitoid Hymenoptera. In a related study comparing feeding generalist and specialist beetles, Farris and Roberts (2005) argue that a generalist life style and associated demands on spatial orientation may have represented a selective pressure for the evolution of large MB calyces. The results by Dacks and Nighorn (2011) and Rössler and Zube (2011) suggest that a dual olfactory pathway in some of the basal plant-feeding Hymenoptera most likely was present before the appearance of large doubled MB calyces and high numbers of olfactory glomeruli. Taking this into account, it is tempting to speculate that the presence of a dual olfactory pathway, large numbers of olfactory glomeruli (Kelber et al. 2009) together with elaborate doubled MB calyces represents a combination of pre-adaptations promoting the emergence of social behavior in Hymenoptera. Further comparative anatomical and functional studies between species within the plant-feeding Symphyta, parasitoid Hymenoptera and closely related solitary versus social Hymenoptera are important to look more deeply into this possibility.

Another important aspect is that comparison between males and females revealed a striking sex-specific difference in the dual olfactory pathway in the ants *C. floridanus* (Zube and Rössler 2008) and *Camponotus japanicus* (Nakanishi et al. 2009, 2010). Interestingly, In *C. floridanus*, the number of m-APT associated glomeruli in the AL is significantly reduced (by ~45 %) in males compared to females (Zube and Rössler 2008). Studies by Sandoz (2006) and Nishino et al. (2009) as well as ongoing studies in our lab (Kropf et al. 2012) indicate that a similar reduction is present in honeybee drones. Interestingly, the reduction of the m-APT associated glomeruli in the male honeybee AL correlates with the lack of sensilla basiconica on male antennae (Lacher 1964; Nishino et al. 2009). In leaf-cutting ants, axons from ORNs housed in sensilla basiconica project to a distinct AL cluster of glomeruli (T6) (Kelber et al. 2010), and total numbers of glomeruli were also shown to be reduced in males of different ant species (Hoyer et al. 2005; Kuebler et al. 2010; Nakanishi et al. 2010).

The sex-specific differences in the organization of the olfactory pathway are likely to be controlled by haplo-diploid genetics in Hymenoptera and the Hymenoptera-specific mode of sex-determination (Beye et al. 2003; Gempe et al. 2009). This may be used as a future tool for targeted developmental manipulations of the central and peripheral olfactory pathways. Finally, it will be particularly interesting to compare males and females in solitary Hymenoptera regarding these differences in olfactory subsystems.

## Conclusions and outlook

We conclude that differential processing of same odorants along two central olfactory pathways in the honeybee matches the criteria of parallel processing comparable to other sensory systems (e.g., auditory: Yu and Young 2000; Rauschecker and Scott 2009; visual: Livingstone and Hubel 1988; Strausfeld et al. 2006; Paulk et al. 2008; Nassi and Callaway 2009; electrosensory: Metzner and Juranek 1997; somatosensory: Ahissar et al. 2000).

### Parallel olfactory processing: odor quality and temporal coding

The fact that the two olfactory PN populations in the honeybee perform parallel processing by extraction of different parameters of the same odorant stimulus opens up novel ways to think about parallel coding in the three higher-order target areas of the honeybee olfactory system—the medial and lateral MB calyces, and the LH (Figs. 1, 3). The temporal delays of incoming odorant-evoked spike sequences from l- and m-APT PNs via the two opposing delay-line-like neuronal circuits may enhance certain aspects of olfactory coding by employing a coincidence code at the level of KCs (see models in Figs. 1c, 3). We hypothesize that this delay-line-like circuitry might, for example, improve coding and detection of different ratios of odorant intensities in complex mixtures such as natural odorants, pheromones, and multi-component cuticular recognition cues.

Within this system, the output of PN activation along both pathways is “checked” at three target points that differ in space and timing of activity (Figs. 1, 3). KCs in the medial and lateral MB calyces that are innervated by axon collaterals of the same PN are likely to transfer slightly different information to the MB lobes as PN spikes arrive with a slight temporal difference (~2 ms) at the two MB calyces (Fig. 3). At the level of the MB lobes, axonal projections from KCs originating in the medial and lateral MB calyces are likely to converge to the same output layer (Strausfeld 2002). Here, activity from both input channels could either be averaged or processed differentially. This might, for example, improve either the accuracy and/or dynamic range for odorant intensity coding. At the level of the LH, convergent input from l- and m-APT PNs on LH postsynaptic neurons could, for example, be used for left–right comparison of incoming olfactory information and/or rapid activation of pre-motor circuits important for fast flight steering commands. As an alternative hypothesis, different features of an olfactory stimulus could be extracted differentially at the three “check points” via specific synaptic circuits that act in the three axonal collaterals of the same PNs. In the same line, the different targets could be under the influence of different neuromodulators. Interestingly, a study in *Drosophila* showed that axon collaterals of axonal branches of ORNs in the ipsi- and contralateral AL significantly differed in the amount of neurotransmitter released (Gaudry et al. 2013). To address whether differential processing of l- and m-APT input takes place at the level of MB calyx and LH, we need more high-resolution circuit analyses at the level of these higher centers and more information about the nature of postsynaptic neurons (see below).

To start to test these hypotheses, an important future step is to obtain simultaneous recordings from PNs and KCs or LH postsynaptic neurons. This should be started using unimolecular odorant stimuli as well as systematic variations in the concentrations of individual compounds and their ratios in mixtures. Another promising approach is to establish temporally defined activation of PNs by selective electrical stimulation of the two PN populations with defined temporal delays and, at the same time, record from KCs in the two MB calyces or the postsynaptic neurons in the LH. This addresses the question whether temporal response properties, in particular synchrony of l- and m-APT PN activities, are relevant for coincidence coding at the level of KCs. The special anatomical features of the dual olfactory pathway in the honeybee provide an ideal substrate for these highly promising approaches to investigate temporal coding in this dual olfactory system.

We also need more precise information on the different KC populations in the MB calyx, especially how they are synaptically connected with PNs from both tracts and whether this provides a potential substrate for dual-tract coincidence coding (Fig. 3). Individual KCs could either receive input from only l- or m-APT PNs, or convergent input from both information streams. KC dendritic morphologies obtained from Golgi studies (Strausfeld 2002) and their comparison with l- and m-APT PN target areas (Kirschner et al. 2006) indicates that, in principle, all three scenarios are possible. Physiological studies have shown that KCs may serve as coincidence detectors promoting sparse coding (Perez-Orive et al. 2002, 2004; Gupta and Stopfer 2012), and in situ patch-clamp analyses in the cockroach revealed that intrinsic membrane and ion-channel properties of KCs are well suited for coincidence coding (Demmer and Kloppenburg 2009).

### Possible causes for differential processing along two olfactory information streams

It is still an open question whether the differences in response properties between l- and m-APT PNs as shown in Brill et al. (2013) are the result of differential sensory input from ORNs, differences in local AL processing via local interneurons, and/or differences in intrinsic properties of the two uniglomerular PN populations. Recent physiological studies suggest that lateral inhibition and gain control mechanisms in the AL are mediated by different types of local interneurons (Assisi et al. 2011, 2012; Martin et al. 2011; Wilson 2011). A modeling study indicates that this allows variable tuning of odorant specificity and concentration dependence in honeybee PNs (Schmuker et al. 2011; Nawrot 2012). This would mean that m-APT PNs undergo stronger lateral inhibition and gain control mechanisms compared to the more broadly tuned l-APT PNs. To evaluate whether differences in intrinsic properties of the two PN populations contribute to differences in odorant processing, in situ patch-clamp analyses of ion-channel composition and synaptic currents in l- and m-APT PNs in the honeybee may provide answers in the future.

The neurotransmitters and modulators of both systems are only partly known. Whereas acetylcholine was shown to be a neurotransmitter of m-APT PNs (Kreissl and Bicker 1989; Barbara et al. 2005, 2008), we still do not know the neurotransmitter employed by l-APT PNs. Furthermore, innervation of m- and l-APT associated glomeruli by serotonergic neurons was shown to be differentially distributed in the AL of the ant *C. floridanus* (Zube and Rössler 2008). It was mostly absent in m-APT associated glomeruli, but prominent in l-APT associated glomeruli. A similar distribution across AL glomeruli was also shown for other ants of the genus *Camponotus* (Dacks et al. 2006; Tsuji et al. 2007). An obvious differential distribution of serotonergic innervation was not found in the honeybee, but it seems worthwhile to look for other neuromodulators that may provide differential influences on processing in the two AL hemilobes (Galizia and Rössler 2010; Galizia and Kreissl 2012).

### Higher-order processing in microcircuits of the mushroom bodies

We also need more information about the functional properties and plasticity at the PN-output side, in particular in PN–KC microcircuits (microglomeruli, MG) in the l- and m-APT target regions of MB calyx. A recent study by Groh et al. (2012) showed that the age- and task-related increase in synaptic divergence in PN–KC synapses was higher in l-APT associated MG compared to those associated with the m-APT. This fits well with the finding that l-APT PNs have broader odorant–response profiles than m-APT PNs and, therefore, are likely to provide higher activation rates to KCs in the course of behavioral development, especially during the transition from nurse bees to foragers.

Individual PN boutons may have synaptic contacts to as much as ~140 postsynaptic profiles, most of them KC dendritic spines (Groh et al. 2012). First estimates of the total numbers of PN–KC synaptic contacts based on serial electron microscopy reconstructions of MG in the MB calyces range around 700,000 PN boutons with a total of ~130 million postsynaptic contacts extrapolated to all four MB calyces (Groh et al. 2012). These numbers suggest an enormous space for synaptic plasticity, which is another area for future exploration of differences between both olfactory information streams (Rössler and Groh 2012). A recent study by Hourcade et al. (2010) showed that the formation of long-term olfactory memory is associated with structural synaptic plasticity in PN–KC synaptic boutons of the olfactory subregions (lip) in the MB calyx. It remained unclear whether certain types of PNs were preferentially affected and whether m- and l-APT PNs may differ in the degree of learning-related plasticity as it was suggested earlier by Peele et al. (2006). In a related context, a recent study by Riffell et al. (2013) in *Manduca sexta* suggests that olfactory stimuli are processed through two olfactory channels, one involving an innate basis and the other learned associations.

### Evolution and functional implications of a dual olfactory pathway in Hymenoptera

The evolution of the dual pathway within the Hymenoptera certainly represents an exciting field for future comparative analyses. A general problem, however, is that the studies, so far, have demonstrated correlations between neuroanatomical traits, phylogenetic relationships, and life styles (e.g., plant feeding, parasitoidism, social) rather than causal relationships like the ability to detect and process certain odorants, to perform sophisticated olfactory-guided behaviors, or the ability for elaborated olfactory communication. Future studies using lesion experiments, pharmacological tools, manipulation by RNA interference, and/or developmental manipulations combined with functional or behavioral studies will be important to understand the physiological mechanisms and causal relationships. In the same line, comparative studies of physiological and behavioral differences between males and females may be elusive to understand the adaptive function of a dual olfactory pathway in Hymenoptera. Behavioral choice or orientation experiments, the well-established classical conditioning paradigm using the proboscis extension response (PER), or other experimental approaches to olfactory learning and memory are promising ways to investigate the functional role of a dual olfactory pathway (e.g., Menzel and Giurfa 2001; Sandoz 2011; Giurfa and Sandoz 2012; Matsumoto et al. 2012; Menzel 2012). However, it is important to mention that the use of the PER may be limited as, for example, it is not elicited in Megachilid bees (Vorel and Pitts-Singer 2010).

The wealth of information we already have from studies on the neuroanatomy, sex-specificity, and evolution of the dual olfactory pathway in Hymenoptera together with recent progress in neurophysiological studies on the honeybee dual olfactory pathway provide exceptional opportunities for future studies aiming at understanding fundamental mechanisms of parallel olfactory processing and general aspects of sensory coding and perception.

## Electronic supplementary material

Below is the link to the electronic supplementary material.

Supplementary material 1 (PDF 25 kb)

## Abbreviations

AL: Antennal lobe
APT: Antennal-lobe protocerebral tract
LH: Lateral horn
MB: Mushroom body
ORN: Olfactory receptor neuron
PER: Proboscis extension response
PN: Projection neuron
T1–T4: Olfactory receptor neuron sensory-input tracts 1–4

## References

[CR1] Abel R, Rybak J, Menzel R (2001). Structure and response patterns of olfactory interneurons in the honeybee, *Apis mellifera*. J Comp Neurol.

[CR2] Ahissar E, Sosnik R, Haidarliu S (2000). Transformation from temporal to rate coding in a somatosensory thalamocortical pathway. Nature.

[CR3] Assisi C, Stopfer M, Bazhenov M (2011). Using the structure of inhibitory networks to unravel mechanisms of spatiotemporal patterning. Neuron.

[CR4] Assisi C, Stopfer M, Bazhenov M (2012). Excitatory local interneurons enhance tuning of sensory information. PLoS Comput Biol.

[CR5] Barbara GS, Zube C, Rybak J, Gauthier M, Grünewald B (2005). Acetylcholine, GABA and glutamate induce ionic currents in cultured antennal lobe neurons of the honeybee, *Apis mellifera*. J Comp Physiol A.

[CR6] Barbara GS, Grünewald B, Paute S, Gauthier M, Raymond-Delpech V (2008). Study of nicotinic acetylcholine receptors on cultured antennal lobe neurones from adult honeybee brains. Invert Neurosci.

[CR7] Beye M, Hasselmann M, Fondrk MK, Page RE, Omholt SW (2003). The gene csd is the primary signal for sexual development in the honeybee and encodes an SR-type protein. Cell.

[CR8] Brandstaetter AS, Kleineidam CJ (2011). Distributed representation of social odors indicates parallel processing in the antennal lobe of ants. J Neurophysiol.

[CR9] Brill MF, Rosenbaum T, Reus I, Kleineidam CJ, Nawrot MP, Rössler W (2013). Parallel processing via a dual olfactory pathway in the honeybee. J Neurosci.

[CR10] Callaway EM (2005). Structure and function of parallel pathways in the primate early visual system. J Neurophysiol.

[CR11] Carcaud J, Hill T, Giurfa M, Sandoz J-C (2012). Differential coding by two olfactory subsystems in the honeybee brain. J Neurophysiol.

[CR12] Cassenaer S, Laurent G (2007). Hebbian STDP in mushroom bodies facilitates the synchronous flow of olfactory information in locusts. Nature.

[CR13] Chen Y-C, Mishra D, Schmitt L, Schmuker M, Gerber B (2011). A behavioral odor similarity “space” in larval *Drosophila*. Chem Senses.

[CR14] Dacks AM, Nighorn AJ (2011). The organization of the antennal lobe correlates not only with phylogenetic relationship, but also life history: a basal hymenopteran as exemplar. Chem Senses.

[CR15] Dacks AM, Christensen TA, Hildebrand JG (2006). Phylogeny of a serotonin-immunoreactive neuron in the primary olfactory center of the insect brain. J Comp Neurol.

[CR16] Dacks AM, Reisemann CE, Paulk AC, Nighorn AJ (2010). Histamine-immunoreactive local neurons in the antennal lobes of the Hymenoptera. J Comp Neurol.

[CR17] Demmer H, Kloppenburg P (2009). Intrinsic membrane properties and inhibitory synaptic input of kenyon cells as mechanisms for sparse coding?. J Neurophysiol.

[CR18] Farris SM, Roberts NS (2005). Coevolution of generalist feeding ecologies and gyrencephalic mushroom bodies in insects. Proc Natl Acad Sci USA.

[CR19] Farris SM, Schulmeister S (2011). Parasitoidism, not sociality, is associated with the evolution of elaborate mushroom bodies in the brains of hymenopteran insects. Proc R Soc B Biol Sci.

[CR20] Fischbach K-F, Dittrich APM (1989). The optic lobe of *Drosophila melanogaster*. I. A Golgi analysis of wild-type structure. Cell Tissue Res.

[CR21] Fukunaga I, Berning M, Kollo M, Schmaltz A, Schaefer AT (2012). Two distinct channels of olfactory bulb output. Neuron.

[CR22] Galizia CG, Kreissl S, Eisenhardt D, Giurfa M, Galizia CG (2012). Neuropeptides in honey bees. Honeybee neurobiology and behavior—a tribute to Randolf Menzel.

[CR23] Galizia CG, Rössler W (2010). Parallel olfactory systems in insects: anatomy and function. Annu Rev Entomol.

[CR24] Galizia CG, McIlwrath SL, Menzel R (1999). A digital three-dimensional atlas of the honeybee antennal lobe based on optical sections acquired by confocal microscopy. Cell Tissue Res.

[CR25] Galizia CG, Franke T, Menzel R, Sandoz J-C (2012). Optical imaging of concealed brain activity using a gold mirror in honeybees. J Insect Physiol.

[CR26] Gaudry Q, Hong EJ, Kain J, De Bivort BL, Wilson RI (2013). Asymmetric neurotransmitter release enables rapid odour lateralization in *Drosophila*. Nature.

[CR27] Gempe T (2009). Sex determination in honeybees: two separate mechanisms induce and maintain the female pathway. PLoS Biol.

[CR28] Giurfa M, Sandoz J-C (2012). Invertebrate learning and memory: fifty years of olfactory conditioning of the proboscis extension response in honeybees. Learn Mem.

[CR29] Groh C, Rössler W (2008). Caste-specific postembryonic development of primary and secondary olfactory centers in the female honeybee brain. Arthropod Struct Dev.

[CR30] Groh C, Lu Z, Meinertzhagen IA, Rössler W (2012). Age-related plasticity in the synaptic ultrastructure of neurons in the mushroom body calyx of the adult honeybee *Apis mellifera*. J Comp Neurol.

[CR31] Grünewald B (1999). Physiological properties and response modulations of mushroom body feedback neurons during olfactory learning in the honeybee, *Apis mellifera*. J Comp Physiol A.

[CR32] Grünewald B, Eisenhardt D, Giurfa M, Galizia CG (2012). Cellular physiology of the honey bee brain. Honeybee neurobiology and behavior—a tribute to Randolf Menzel.

[CR33] Guerrieri F, Schubert M, Sandoz J, Giurfa M (2005). Perceptual and neural olfactory similarity in honeybees. PLoS Biol.

[CR34] Gupta N, Stopfer M (2012). Functional analysis of a higher olfactory center, the lateral horn. J Neurosci.

[CR35] Haddad R, Khan R, Takahashi YK, Mori K, Harel D, Sobel N (2008). A metric for odorant comparison. Nat Methods.

[CR36] Haddad R, Weiss T, Khan R, Nadler B, Mandairon N, Bensafi M, Schneidman E, Sobel N (2010). Global features of neural activity in the olfactory system form a parallel code that predicts olfactory behavior and perception. J Neurosci.

[CR37] Hansson BS, Stensmyr MC (2011). Evolution of insect olfaction. Neuron.

[CR38] Helversen D, Helversen O (1995). Acoustic pattern recognition and orientation in orthopteran insects: parallel or serial processing?. J Comp Physiol A.

[CR39] Himmelreich S, Grünewald B (2012). Cellular physiology of olfactory learning in the honeybee brain. Apidologie.

[CR40] Hölldobler B (1999). Multimodal signals in ant communication. J Comp Physol A.

[CR41] Hölldobler B, Wilson EO (1990). The ants.

[CR42] Hourcade B, Muenz TS, Sandoz J-C, Rössler W, Devaud J-M (2010). Long-term memory leads to synaptic reorganization in the mushroom bodies: a memory trace in the insect brain?. J Neurosci.

[CR43] Hoyer SC, Liebig J, Rössler W (2005). Biogenic amines in the ponerine ant *Harpegnathos saltator*: serotonin and dopamine immunoreactivity in the brain. Arthropod Struct Dev.

[CR44] Igarashi KM, Ieki N, An M, Yamaguchi Y, Nagayama S, Kobayakawa K, Kobayakawa R, Tanifuji M, Sakano H, Chen WR, Mori K (2012). Parallel mitral and tufted cell pathways route distinct odor information to different targets in the olfactory cortex. J Neurosci.

[CR45] Ito I, Bazhenov M, Ong RC, Raman B, Stopfer M (2009). Frequency transitions in odor-evoked neural oscillations. Neuron.

[CR46] Kelber C, Rössler W, Kleineidam CJ (2006). Multiple olfactory receptor neurons and their axonal projections in the antennal lobe of the honeybee *Apis mellifera*. J Comp Neurol.

[CR47] Kelber C, Rössler W, Roces F, Kleineidam CJ (2009). The antennal lobes of fungus-growing ants (*Attini*): neuroanatomical traits and evolutionary trends. Brain Behav Evol.

[CR48] Kelber C, Rössler W, Kleineidam CJ (2010). Phenotypic plasticity in number of glomeruli and sensory innervation of the antennal lobe in leaf-cutting ant workers (*A. vollenweideri*). Dev Neurobiol.

[CR49] Kirschner S, Kleineidam CJ, Zube C, Rybak J, Grünewald B, Rössler W (2006). Dual olfactory pathway in the honeybee, *Apis mellifera*. J Comp Neurol.

[CR50] Knudsen EI, Du Lac S, Esterly SD (1987). Computational maps in the brain. Annu Rev Neurosci.

[CR51] Kreissl S, Bicker G (1989). Histochemistry of acetylcholinesterase and immunocytochemistry of an acetylcholine receptor-like antigen in the brain of the honeybee. J Comp Neurol.

[CR52] Krofczik S (2007) The honeybee olfactory system: 3-D anatomy, physiology and plasticity. Dissertation, Free University of Berlin

[CR53] Krofczik S, Menzel R, Nawrot MP (2008). Rapid odor processing in the honeybee antennal lobe network. Front Comput Neurosci.

[CR54] Kropf J, Bieringer K, Kelber C, Rössler W (2012). Olfactory subsystems in the honeybee: sensory supply and sex-specifity. Front Behav Neurosci. Conference Abstract: Tenth International Congress of Neuroethology. doi:10.3389/conf.fnbeh.2012.27.00239

[CR55] Kuebler LS, Kelber C, Kleineidam CJ (2010). Distinct antennal lobe phenotypes in the leaf-cutting ant (*Atta vollenweideri*). J Comp Neurol.

[CR56] Lacher V (1964). Elektrophysiologische Untersuchungen an einzelnen Rezeptoren für Geruch, Kohlendioxid, Luftfeuchtigkeit und Temperatur auf den Antennen der Arbeiterbiene und der Drohne (*Apis mellifera* L.). Z vergl Physiol.

[CR57] Laurent G (2002). Olfactory network dynamics and the coding of multidimensional signals. Nat Rev Neurosci.

[CR58] Laurent G, Wehr M, Davidowitz H (1996). Temporal representations of odors in an olfactory network. J Neurosci.

[CR59] Lei H, Christensen TA, Hildebrand JG (2002). Local inhibition modulates odor-evoked synchronization of glomerulus-specific output neurons. Nat Neurosci.

[CR60] Lennie P, Movshon JA (2005). Coding of color and form in the geniculostriate visual pathway (invited review). J Opt Soc Am A.

[CR61] Livingstone M, Hubel D (1988). Segregation of form, color, movement, and depth: anatomy, physiology, and perception. Science.

[CR62] Martin JP, Beyerlein A, Dacks AM, Reisenman CE, Riffell JA, Lei H, Hildebrand JG (2011). The neurobiology of insect olfaction: sensory processing in a comparative context. Prog Neurobiol.

[CR63] Matsumoto Y, Menzel R, Sandoz J-C, Giurfa M (2012). Revisiting olfactory classical conditioning of the proboscis extension response in honey bees: a step toward standardized procedures. J Neurosci Methods.

[CR64] Meier R, Egert U, Aertsen A, Nawrot MP (2008). FIND—a unified frame-work for neural data analysis. Neural Netw.

[CR65] Menzel R (2012). The honeybee as a model for understanding the basis of cognition. Nat Rev Neurosci.

[CR66] Menzel R, Giurfa M (2001). Cognitive architecture of a mini-brain: the honeybee. Trends Cogn Sci.

[CR67] Merigan WH, Maunsell JHR (1993). How parallel are the primate visual pathways?. Annu Rev Neurosci.

[CR68] Metzner W, Juranek J (1997). A sensory brain map for each behavior?. Proc Nat Acad Sci USA.

[CR69] Milner AD, Goodale MA (2008). Two visual systems re-viewed. Neuropsychologia.

[CR70] Mishkin M, Ungerleider LG, Macko KA (1983). Object vision and spatial vision: two cortical pathways. Trends Neurosci.

[CR71] Mizunami M, Okada R, Li Y, Strausfeld NJ (1998). Mushroom bodies of the cockroach: activity and identities of neurons. J Comp Neurol.

[CR72] Mobbs P (1982). The brain of the honeybee *Apis mellifera*. I. The connections and spatial organization of the mushroom bodies. Philos Trans R Soc Lond B Biol Sci.

[CR73] Müller D, Abel R, Brandt R, Zöckler M, Menzel R (2002). Differential parallel processing of olfactory information in the honeybee, *Apis mellifera* L.. J Comp Physiol A.

[CR74] Nakanishi A, Nishino H, Watanabe H, Yokohari F, Nishikawa M (2009). Sex-specific antennal sensory system in the ant *Camponotus japonicus*: structure and distribution of sensilla on the flagellum. Cell Tissue Res.

[CR75] Nakanishi A, Nishino H, Watanabe H, Yokohari F, Nishikawa M (2010). Sex-specific antennal sensory system in the ant *Camponotus japonicus*: glomerular organizations of antennal lobes. J Comp Neurol.

[CR76] Nassi JJ, Callaway EM (2009). Parallel processing strategies of the primate visual system. Nat Rev Neurosci.

[CR77] Nawrot MP (2012). Dynamics of sensory processing in the dual olfactory pathway of the honeybee. Apidologie.

[CR78] Nawrot MP, Aertsen A, Rotter S (2003). Elimination of response latency variability in neuronal spike trains. Biol Cybern.

[CR79] Nishikawa M, Watanabe H, Yokohari F (2012). Higher brain centers for social tasks in worker ants, *Camponotus japonicus*. J Comp Neurol.

[CR80] Nishino H, Nishikawa M, Mizunami M, Yokohari F (2009). Functional and topographic segregation of glomeruli revealed by local staining of antennal sensory neurons in the honeybee *Apis mellifera*. J Comp Neurol.

[CR81] Nishino H, Yoritsune A, Mizunami M (2010). Postembryonic development of sexually dimorphic glomeruli and related interneurons in the cockroach *Periplaneta americana*. Neurosci Lett.

[CR82] Nishino H, Iwasaki M, Kamimura I, Mizunami M (2012). Divergent and convergent projections to the two parallel olfactory centers from two neighboring, pheromone-receptive glomeruli in the male American cockroach. J Comp Neurol.

[CR83] Oleskevich S, Clements JD, Srinivasan MV (1997). Long-term synaptic plasticity in the honeybee. J Neurophysiol.

[CR84] Paulk AC, Phillips-Portillo J, Dacks AM, Fellous J-M, Gronenberg W (2008). The processing of color, motion, and stimulus timing are anatomically segregated in the bumblebee brain. J Neurosci.

[CR85] Paulk AC, Dacks AM, Gronenberg W (2009). Color processing in the medulla of the bumblebee (Apidae: *Bombus impatiens*). J Comp Neurol.

[CR86] Payton CA, Wilson DA, Wesson DW (2012). Parallel odor processing by two anatomically distinct olfactory bulb target structures. PLoS One.

[CR87] Peele P, Ditzen M, Menzel R, Galizia CG (2006). Appetitive odor learning does not change olfactory coding in a subpopulation of honeybee antennal lobe neurons. J Comp Physiol A.

[CR88] Perez-Orive J, Mazor O, Turner GC, Cassenaer S, Wilson RI, Laurent G (2002). Oscillations and sparsening of odor representations in the mushroom body. Science.

[CR89] Perez-Orive J, Bazhenov M, Laurent G (2004). Intrinsic and circuit properties favor coincidence detection for decoding oscillatory input. J Neurosci.

[CR90] Rauschecker JP, Scott SK (2009). Maps and streams in the auditory cortex: nonhuman primates illuminate human speech processing. Nat Neurosci.

[CR91] Ribi WA, Scheel M (1981). The second and third optic ganglia of the worker bee: Golgi studies of the neuronal elements in the medulla and lobula. Cell Tissue Res.

[CR92] Riffel JA, Lei H, Abrell L, Hildebrand JG (2013). Neural basis of a pollinator*’*s buffet: olfactory specialization and learning in *Manduca sexta*. Science.

[CR93] Riffell JA, Lei H, Christensen TA, Hildebrand JG (2009). Characterization and coding of behaviorally significant odor mixtures. Curr Biol.

[CR94] Riffell JA, Lei H, Hildebrand JG (2009). Neural correlates of behavior in the moth *Manduca sexta* in response to complex odors. Proc Nat Acad Sci USA.

[CR95] Rössler W, Groh C, Eisenhardt D, Giurfa M, Galizia CG (2012). Plasticity of synaptic microcircuits in the mushroom-body calyx of the honey bee. Honeybee neurobiology and behavior—a tribute to Randolf Menzel.

[CR96] Rössler W, Zube C (2011). Dual olfactory pathway in Hymenoptera: evolutionary insights from comparative studies. Arthropod Struct Dev.

[CR97] Rybak J, Eisenhardt D, Giurfa M, Galizia CG (2012). The digital honey bee brain atlas. Honeybee neurobiology and behavior—a tribute to Randolf Menzel.

[CR98] Sandoz J-C (2006). Odour-evoked responses to queen pheromone components and to plant odours using optical imaging in the antennal lobe of the honey bee drone *Apis mellifera* L.. J Exp Biol.

[CR99] Sandoz J (2011). Behavioral and neurophysiological study of olfactory perception and learning in honeybees. Front Syst Neurosci.

[CR100] Sandoz J-C, Eisenhardt D, Giurfa M, Galizia CG (2012). Olfaction in honey bees: from molecules to behavior. Honeybee neurobiology and behavior—a tribute to Randolf Menzel.

[CR101] Sandoz J, Deisig N, De Brito Sanchez MG, Giurfa M (2007). Understanding the logics of pheromone processing in the honeybee brain: from labeled-lines to across-fiber patterns. Front Behav Neurosci.

[CR102] Schlief ML, Wilson RI (2007). Olfactory processing and behavior downstream from highly selective receptor neurons. Nat Neurosci.

[CR103] Schmuker M, Schneider G (2007). Processing and classification of chemical data inspired by insect olfaction. Proc Nat Acad Sci USA.

[CR104] Schmuker M, Yamagata N, Nawrot Martin P, Menzel R (2011). Parallel representation of stimulus identity and intensity in a dual pathway model inspired by the olfactory system of the honeybee. Front Neuroeng.

[CR105] Seeley TD (1995). Whisdom of the hive: the social physiology of honey bee colonies.

[CR106] Slessor KN, Winston ML, Le Conte Y (2005). Pheromone communication in the honeybee (*Apis mellifera* L.). J Chem Ecol.

[CR107] Smith AA, Hölldobler B, Liebig Jürgen (2009). Cuticular hydrocarbons reliably identify cheaters and allow enforcement of altruism in a social insect. Curr Biol.

[CR108] Stopfer M, Bhagavan S, Smith BH, Laurent G (1997). Impaired odour discrimination on desynchronization of odour-encoding neural assemblies. Nature.

[CR109] Stopfer M, Jayaraman V, Laurent G (2003). Intensity versus identity coding in an olfactory system. Neuron.

[CR110] Strausfeld NJ (2002). Organization of the honey bee mushroom body: representation of the calyx within the vertical and gamma lobes. J Comp Neurol.

[CR111] Strausfeld NJ, Douglass JK, Campbell H, Higgins C, Warrant E, Nilsson D-E (2006). Parallel processing in the optic lobes of flies and the occurrence of motion computing circuits. Invertebrate vision.

[CR112] Strube-Bloss MF, Nawrot Martin P, Menzel R (2011). Mushroom body output neurons encode odor-reward associations. J Neurosci.

[CR113] Strube-Bloss MF, Herrera-Valdez MA, Smith BH (2012). Ensemble response in mushroom body output neurons of the honey bee outpaces spatiotemporal odor processing two synapses earlier in the antennal lobe. PLoS One.

[CR114] Sun X-J, Fonta C, Masson C (1993). Odour quality processing by bee antennal lobe interneurones. Chem Senses.

[CR115] Tabuchi M, Inoue S, Kanzaki R, Nakatani K (2012). Whole-cell recording from Kenyon cells in silkmoths. Neurosci Lett.

[CR116] Tsuji E, Aonuma H, Yokohari F, Nishikawa M (2007). Serotonin-immunoreactive neurons in the antennal sensory system of the brain in the Carpenter Ant, *Camponotus japonicus*. Zool Sci.

[CR117] Vorel CA, Pitts-Singer TL (2010). Proboscis extension reflex not elicited in megachilid bees. J Kansas Entomol Soc.

[CR118] Wehr M, Laurent G (1996). Odour encoding by temporal sequences of firing in oscillating neural assemblies. Nature.

[CR119] Wilson RI (2011). Understanding the functional consequences of synaptic specialization: insight from the *Drosophila* antennal lobe. Curr Opin Neurobiol.

[CR120] Winston ML (1987). The biology of the honey bee.

[CR121] Wyatt TD (2010). Pheromones and signature mixtures: defining species-wide signals and variable cues for identity in both invertebrates and vertebrates. J Comp Physiol A.

[CR122] Yamagata N, Schmuker M, Szyszka P, Mizunami M, Menzel R (2009). Differential odor processing in two olfactory pathways in the honeybee. Front Syst Neurosci.

[CR123] Young ED (1998). Parallel processing in the nervous system: evidence from sensory maps. Proc Nat Acad Sci USA.

[CR124] Yu JJ, Young ED (2000). Linear and nonlinear pathways of spectral information transmission in the cochlear nucleus. Proc Nat Acad Sci USA.

[CR125] Zube C, Rössler W (2008). Caste- and sex-specific adaptations within the olfactory pathway in the brain of the ant *Camponotus floridanus*. Arthropod Struct Dev.

[CR126] Zube C, Kleineidam CJ, Kirschner S, Neef J, Rössler W (2008). Organization of the olfactory pathway and odor processing in the antennal lobe of the ant *Camponotus floridanus*. J Comp Neurol.

