# Supplementary material for: Parallel processing in the honeybee olfactory pathway: structure, function, and evolution
Source: J Comp Physiol A Neuroethol Sens Neural Behav Physiol. 2013 Apr 23;199(11):981–96. doi: 10.1007/s00359-013-0821-y (PMC3824823; doi:10.1007/s00359-013-0821-y)
Supplement: Supplementary file 1 — Supplementary material 1 (PDF 25 kb) [file 359_2013_821_MOESM1_ESM.pdf]

**Parallel processing in the honeybee olfactory pathway: structure, function and evolution**
Journal of Comparative Physiology A
Wolfgang Rössler* and Martin F. Brill
Behavioral Physiology and Sociobiology (Zoology II), Biozentrum,
University of Würzburg, 97074 Würzburg, Germany
* roessler@biozentrum.uni-wuerzburg.de

**Supplemental Table 1:** Comparison of the panels of odorants used in different studies on the dual olfactory pathway of the honeybee referenced in the text. The complete references are listed under the list of references. [1] Further odorants (up to 30) were tested in individual neurons. See also dissertation by Krofczik (2007).

| | recording method electrophysiology [e] or calcium imaging [c] | concentration range [log] | 6al (hexanal) | 7al | 8al | 9al | 1-5ol (1-pentanol | 1-6ol | 1-7ol | 1-8ol | 1-9ol | 2-6ol (2-hexanol) | 2-7ol | 2-8ol | 2-9ol | 2-6one (2-hexanone) | 2-7one | 2-8one | 2-9one | benzaldehyde | carnation | cineol | citral | clove oil | geraniol | geranic acid | isoamyl acetate | lime tree | limonene | linalool | orange oil | peppermint | brood comb | dead bees | honey in water | clean Bees wax | mixed bee's wax |
|---|---|---|---|---|---|---|---|---|---|---|---|---|---|---|---|---|---|---|---|---|---|---|---|---|---|---|---|---|---|---|---|---|---|---|---|---|---|
| **Sun et al. 1993** | e single | -2 | | | | | | | | | | | | | | | x | | | | | | | | x | | x | | | | | | | | | | |
| **Abel et al. 2001** | e single | pure | x | | | | | x | | | | | | | | | | | | | x | | x | | x | | | | | | x | | | | | | |
| **Müller et al. 2002** | e single | pure | x | | | | | x | | x | | | | | | x | x | x | | | | | x | x | x | | x | x | | | x | x | | | | | |
| **[1]Krofczik et al. 2008** | e single | -1 | | | | | | x | | | x | | | | | | x | | | | | | | | | | | | | | | | | | | | |
| **Yamagata et al. 2009** | c | -5 to 0 | x | | x | | | x | | x | | | | x | | | x | x | | | | | | | | | | | | x | | | | | | | |
| **Galizia et al. 2012** | c | (1.79 to 440 µl/ml) | | | x | | | x | | x | x | | | x | | | x | | | x | | x | x | | x | | x | | x | x | | | | | | | |
| **Carcaud et al. 2012** | c | -7 to 0 | x | x | x | x | | x | x | x | x | x | x | x | x | x | x | x | x | | | | | | | | | | | | | | | | | | |
| **Brill et al. 2013** | e multi | -2 | x | | | | x | x | | x | | | x | | | | x | x | | | | | x | x | | x | x | | x | | x | | x | x | x | x | x |
